# The Contribution of Imaging Beyond Clinical Diagnosis, the Ochronosis and Synovio-entheseal Complex Examples

**DOI:** 10.2478/rir-2022-0009

**Published:** 2022-07-06

**Authors:** Giovanni Pacini, Marco Matucci Cerinic

**Affiliations:** 1 Department of Experimental and Clinical Medicine University of Florence and Division of Rheumatology AOUC, Firenze, Italy; 2 Unit of Immunology, Rheumatology, Allergy and Rare diseases (UnIRAR), IRCCS San Raffaele Hospital, Milan, Italy

Ochronosis is the musculoskeletal manifestation of alkaptonuria (AKU), a rare autosomal recessive disorder caused by a mutation of the homogentisate 1,2 dioxygenase (HGD) gene encoding for the HGD enzyme involved in the metabolism of tyrosine and phenylalanine.^[1]^ This metabolic dysfunction leads to increased blood and urinary concentration of homogentisic acid (HGA), a molecule which aggregates in pigmented deposits that can be found in collagenic tissues, particularly in cartilage, fibrocartilage, tendons, and ligaments.^[2, 3]^ Further, HGA pigmented deposition could lead to the formation of urinary stones, ochronotic pigmentation of the sclera, aortic or mitral valve, and darkening of the conchal bowl of the external ear due to ochronosis of the underlying elastic cartilage.^[4]^

Despite being a rare disease, ochronosis has a highly negative impact on the patient's quality of life and, unfortunately, no specific treatment has yet been discovered for this condition.

Therefore, an increasing number of studies are focusing on this rare disease, trying to discover new specific agents aimed to treat this condition.

In their review, Gallagher *et al*.^[5]^ provide substantial insights on the many issues concerning AKU and more specifically, ochronosis. It is interesting to note that Gallagher *et al*.^[5]^ report a pathogenetic insight shared by primary osteoarthritis (OA) and OA associated with ochronosis. In detail, they highlight the role of high-density mineralized protrusions (HDMP) in damaging patients’ cartilage and promoting the development of OA both in patients with ochronosis and those with a common form of OA. The Authors evidenced that HDMP can be easily detected in everyday clinical practice with magnetic resonance imaging (MRI), micro-computed tomography (CT), and scanning electron microscopy. These imaging techniques show that it is possible to detect microanatomical alterations both in very frequent diseases and also in rare diseases, like ochronosis.

In fact, the example provided by ochronosis is not an isolated case in this innovative application of imaging. Similar findings in imaging application have been recently applied to other rheumatic diseases such as spondyloarthritis (SpA) and Systemic Sclerosis (SSc). A large body of evidence has led to the knowledge that SpA and SSc share some clinical manifestations, like sacroiliitis, synovitis, and tenosynovitis as well.^[6, 7]^ Moreover, the use of ultrasound (US) in SSc patients has shown that enthesitis is another clinical feature frequently shared by SSc and SpA. Terenzi *et al*.^[8]^ have demonstrated that these different pathologies share the involvement of the same microanatomical target in enthesitis, that is, the synovio-entheseal complex (SEC). The SEC involvement in SpA related enthesitis has been exhaustively discussed by Benjamin and McGonagle^[9]^, who reported that enthesopathy is commonly associated with alterations of the adjacent bone and synovial tissue. Moreover, SEC involvement in SpA is a clear example of how the application of modern imaging techniques eventually made the study of microanatomic details easier and more accessible to physicians. Previously, the enthesis areas were assessed only by bioptic studies that proved the important anatomical interdependence between adjacent synovial membrane and enthesis themselves.^[10]^ Later, the development of advanced imaging tools, like MRI and US, made the assessment of the “enthesis organ” possible, thus allowing both the diagnosis and follow-up of SpA, as well as giving a better understanding of pathogenetic mechanisms of SpA.^[11, 12]^

In SSc, US has been extensively employed^[13]^ while the SEC involvement has been studied and only recently confirmed. The US assessment of common extensor tendons enthesis in SSc patients clearly shows that it is possible to detect SEC inflammation in these patients as well as those with SpA.^[8]^

Today, the discovery of shared microanatomical targets between common and rare diseases with imaging could have several implications in better understanding pathogenetic mechanisms and consequently, towards finding new therapeutic strategies for these pathologies. In fact, both Gallagher *et al*.^[5]^ and Terenzi *et al*.^[8]^ showed that these common micro-anatomical alterations in two different pathologies could have therapeutic implications. For ochronosis, the discovery of a therapy targeting HDMP could also be useful in OA treatment. Similarly, the discovery of SEC involvement in SSc could lead to the future application of SpA specific target therapies in SSc patients as well, like monoclonal antibodies targeting interleukin-17 and interleukin-12/23 axis.^[14, 15]^

In summary, these studies highlight why the use of advanced imaging should not be limited to diagnosis or follow up of rheumatological diseases alone, but how it could also be used for the study of the pathogenetic mechanisms of musculoskeletal conditions, providing important insights that, only a few years ago, could be obtained only through biopsies. There is a whole world to discover under the surface and imaging represents the right tool to dig deeply into the anatomopathological aspects of musculoskeletal diseases.
